# Severe toxicity induced by accumulation of active sunitinib metabolite in a Japanese patient with renal cell carcinoma: a case report

**DOI:** 10.1186/s13256-016-1185-z

**Published:** 2017-02-01

**Authors:** Shinya Takasaki, Masafumi Kikuchi, Yoshihide Kawasaki, Akihiro Ito, Yoichi Arai, Hiroaki Yamaguchi, Nariyasu Mano

**Affiliations:** 1 Department of Pharmaceutical Sciences, Tohoku University Hospital, 1-1 Seiryo-machi, Aobaku, Sendai, Miyagi 980-8574 Japan; 2 Department of Urology, Tohoku University Hospital, 1-1 Seiryo-machi, Aobaku, Sendai, Miyagi 980-8574 Japan

**Keywords:** Tyrosine kinase inhibitor, Sunitinib, *N*-desethyl sunitinib, Hemodialysis, Therapeutic drug monitoring

## Abstract

**Background:**

Sunitinib is a multi-targeted tyrosine kinase inhibitor that is approved for treatment of renal cell carcinoma as an oral anticancer drug. Therapeutic drug monitoring of total sunitinib (sunitinib and *N*-desethyl sunitinib) is used in our hospital to improve therapeutic efficacy, while preventing adverse effects. Here, we report the first case of a patient with metastatic renal cell carcinoma undergoing hemodialysis and presenting severe adverse events induced by the accumulation of *N*-desethyl sunitinib.

**Case presentation:**

A 60-year-old Japanese man was diagnosed with metastatic renal cell carcinoma requiring hemodialysis. On day 26 of the first cycle of sunitinib therapy, our patient presented grade 3 thrombocytopenia and leukopenia, which required interruption of therapy although the plasma levels of total sunitinib in the patient were less than the effective concentration of 50 ng/mL. The elimination half-life of sunitinib was normal at 50.8 hours, but that of *N*-desethyl sunitinib was an extended 211.4 hours. Moreover, the *N*-desethyl sunitinib/sunitinib trough level ratio was higher than 1.0. We attribute our patient’s severe adverse events to the excessive accumulation of *N*-desethyl sunitinib owing to its delayed excretion. Although the reason for the delayed excretion of *N*-desethyl sunitinib in this patient was unknown, it may have been caused by genetic polymorphisms related to the pharmacokinetics of sunitinib rather than the hemodialysis. In this case, the patient was homozygous for the *ABCG2 421C* allele, but was capable of potentially harboring polymorphisms in other genes, such as *ABCB1*, an efflux pump of sunitinib. In addition, even though there is no clear evidence, urinary excretion of the metabolic products of *N*-desethyl sunitinib could be inhibited by the interaction of transporters such as the organic ion transporter.

**Conclusions:**

The monitoring of not only total sunitinib concentration but also *N*-desethyl sunitinib concentration and their elimination half-lives during sunitinib therapy is recommended to avoid critical adverse events.

## Background

Renal cell carcinoma (RCC) is the most common type of kidney cancer. RCC represents approximately 90% of all renal tumors, and 85% of these RCC consist of clear cell tumors. Recently, many tyrosine kinase inhibitors have been approved for treatment of RCC [1]. Sunitinib, an oral anticancer drug, is a tyrosine kinase inhibitor with multiple targets, including: vascular endothelial growth factor receptors (VEGFRs-1, -2, and -3), platelet-derived growth factor receptors (PDGFRs-α and -β), and the FMS-like tyrosine kinase 3 receptor [2]. Although sunitinib shows a high objective response rate and significantly prolongs the median progression-free survival in the first-line treatment of RCC, sunitinib use has been associated with various adverse events, such as diarrhea, fatigue, vomiting, leukopenia, neutropenia, and thrombocytopenia [3].

Recently, therapeutic drug monitoring (TDM) of anticancer drugs has been reported to improve therapeutic efficacy while preventing the presentation of adverse effects [4]. Sunitinib is metabolized primarily by cytochrome P450 (CYP) 3A4 to *N*-desethyl sunitinib, which shows an activity similar to that of sunitinib [5, 6]. Therefore, TDM of sunitinib is commonly evaluated as total sunitinib (sunitinib and *N*-desethyl sunitinib) in plasma [5]. The effective blood trough concentration of total sunitinib has been reported in the range of 50–100 ng/mL. For sunitinib to inhibit phosphorylation of VEGFR and PDGFR and thus present antitumor activity, a concentration of more than 50 ng/mL total sunitinib is required [2]. In contrast, patients with plasma levels equal to or exceeding 100 ng/mL total sunitinib have frequently developed grade 3 or higher toxicities, such as asthenia and anorexia [7, 8]. The difference between toxicities induced by sunitinib and *N*-desethyl sunitinib is unknown, although sunitinib has been reported to be more dermatotoxic than *N*-desethyl sunitinib is in some patients [9]. Since patients with renal function impairment are usually excluded from clinical trials, pharmacokinetic and pharmacodynamic analyses of sunitinib in such patients are limited. However, a few studies have reported that the pharmacokinetics of sunitinib in patients with severe renal impairment is similar to that of patients with normal renal function [10]. In addition, a case report indicated that the pharmacokinetics of sunitinib and *N*-desethyl sunitinib were not affected by hemodialysis, and sunitinib therapy continued safely [11, 12].

Herein, we report the case of a patient with metastatic RCC undergoing hemodialysis who presented severe adverse events while in treatment with sunitinib. TDM suggests that these events were the result of accumulation of an active sunitinib metabolite even though his total sunitinib levels were below 50 ng/mL.

## Case presentation

A 60-year-old Japanese man was diagnosed with RCC 11 years ago. Initial treatment consisted of a partial left nephrectomy for clear cell carcinoma (pT2 pV0 pM0). Eight years later, our patient presented a cystic kidney and was referred to hemodialysis. Subsequently, our patient presented RCC with bone and brain metastasis and initiated sunitinib therapy. Our patient was prescribed 25 mg sunitinib once daily for 4 weeks of a 6-week cycle. His Eastern Cooperative Oncology Group (ECOG) score was 1. Our patient reported suffering insomnia and bone pain caused by bone metastasis.

**Fig. 1:**
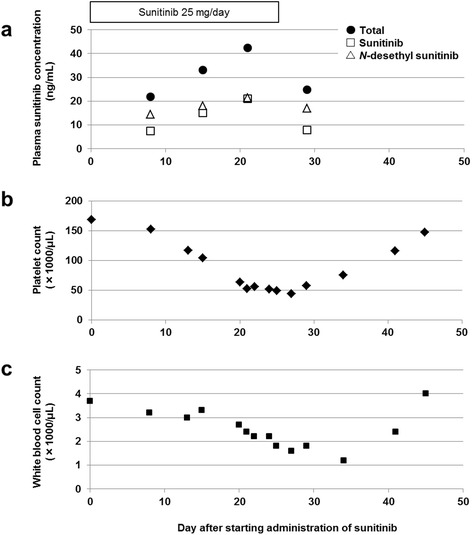
Change in (**a**) plasma concentration of total (sunitinib + *N*-desethyl sunitinib), sunitinib and *N*-desethyl sunitinib, (**b**) platelet count, and (**c**) white blood cell count in the patient after starting sunitinib therapy

One week after starting sunitinib therapy, our patient experienced hand-foot syndrome and grade 1 hypertension as per the Common Terminology Criteria for Adverse Events (version 4.0). On day 26 of first cycle, sunitinib administration was interrupted because our patient presented adverse events of grade 3 thrombocytopenia (platelet count, 49,000/μL) and leukopenia (white blood cell count, 1600/μL) (Fig. 1b and c). On day 21, the concentrations of sunitinib, *N*-desethyl sunitinib and trough level ratio (*N*-desethyl sunitinib/sunitinib) at steady state were 21.1 ng/mL, 21.4 ng/mL, and 1.0, respectively, and 7.9 ng/mL, 16.9 ng/mL, and 2.1, respectively, 4 days after sunitinib withdrawal, as shown in Fig. 1a. Moreover, the elimination half-lives of sunitinib and *N*-desethyl sunitinib were 50.8 hours and 211.4 hours, respectively. After recovering from the hematotoxic events described above, our patient was prescribed axitinib, a second-line drug for metastatic RCC.

With the patient’s written informed consent, blood samples were collected immediately before administration of sunitinib. Plasma levels of sunitinib and *N*-desethyl sunitinib were measured using liquid chromatography-tandem mass spectrometry (LC-MS/MS). The LC-MS/MS assay was carried out using a liquid chromatography system consisting of a Nexera chromatograph (Shimadzu, Kyoto, Japan) coupled to an API 5000 triple-quadrupole tandem mass spectrometer (AB Sciex, Framingham, MA, USA). Our patient was administered benzodiazepines for insomnia, and acetaminophen and tramadol for bone pain. Lansoprazole was started at the same time as the sunitinib treatment, but he continued taking it even after discontinuation of sunitinib. His body weight and body mass index at the start of treatment were 64.3 kg and 23.3 kg/m^2^, respectively. The laboratory analyses while he was undergoing sunitinib therapy yielded the following results: aspartate aminotransferase, 18.7 ± 2.2 U/L; alanine aminotransferase, 14.7 ± 2.0 U/L. Our patient received hemodialysis treatment for 4 hours, three times a week, through a polysulfone dialyzer (VPS-15HA); the blood flow rate was constant at 200 mL/min and the dialysis flow rate was 550 mL/min. In addition, his serum creatinine was 7.7 mg/dL before sunitinib treatment started, 7.0 mg/dL immediately before interruption and 8.8 mg/dL after 1 week of discontinuation.

Genomic deoxyribonucleic acid (DNA) was extracted from the blood and genotyped using direct sequencing of the ATP-binding cassette subfamily G member 2 (*ABCG2*). His genotype was wild type for the *ABCG2 421*C > A polymorphism.

## Discussion

This is the first case in which the accumulation of *N*-desethyl sunitinib was reported to cause serious adverse events, even when the total sunitinib concentration was under the reported trough value of 50 ng/mL. Our data suggest that in this patient, *N*-desethyl sunitinib accumulated because of delayed excretion, resulting in the described adverse effects. The patient’s *N*-desethyl sunitinib plasma levels (21.4 ng/mL) while taking 25 mg sunitinib were higher than the steady-state levels (18.8 ng/mL) of patients administered 50 mg sunitinib daily [7]. The elimination half-lives of sunitinib and *N*-desethyl sunitinib have been reported as 70 hours and 111 hours, respectively, in patients with end-stage renal disease requiring hemodialysis [10]; the corresponding half-lives of this patient were 51 hours and 211 hours. Moreover, the reported trough level ratio (*N*-desethyl sunitinib/sunitinib) is 0.43 [7], but was above 1.0 in this patient. In addition, it has been reported that thrombocytopenia is more frequently associated with *N*-desethyl sunitinib rather than sunitinib [13]. On the basis of these data, we conclude that the cause of the grade 3 hematologic toxicities was the accumulation of *N*-desethyl sunitinib. Despite the fact that the use of concomitant medications, health supplements, and grapefruit have been found to affect the main metabolic enzyme of sunitinib, CYP 3A4, our patient did not consume them; therefore, it was considered unlikely that the accumulation of *N*-desethyl sunitinib was the result of any of these factors. Although the reason for the delayed excretion of *N*-desethyl sunitinib in this patient was unknown, we suggest it was caused by genetic polymorphisms affecting the pharmacokinetics of sunitinib rather than by the hemodialysis. It has been reported that the gene polymorphism in *ABCG2 421C* > *A* can induce severe toxicity by delaying the excretion of sunitinib [14], but the patient in this case had a wild-type *ABCG2*. However, there may be other gene polymorphisms, such as those described for ATP-binding cassette subfamily B member 1, which function as an efflux pump of sunitinib and can thus affect the clinical outcome [15]. Furthermore, *N*-desethyl sunitinib is further metabolized and excreted in the urine [16, 17]. This excretion process can be inhibited by the interaction with transporters such as the organic ion transporter. In addition, previous research has shown that the incidence of severe hematological toxicities is demonstrably higher in Japanese patients than in other populations, but that the pharmacokinetic data from Japanese patients is similar to that of Caucasians [18]. Thus, ethnic differences in sunitinib-induced hematologic toxicities could be attributed to factors other than the pharmacokinetic genetic background.

Although the evidence for use of TDM of sunitinib in a clinical setting is limited, this case report demonstrates that TDM can be useful for predicting and avoiding severe adverse events. Further studies would be needed to select patients suitable for TDM with gene polymorphisms, severe adverse events, and no antitumor effects.

## Conclusions

The monitoring of not only total sunitinib concentration but also *N*-desethyl sunitinib concentration, as well as their elimination half-lives during sunitinib therapy is recommended to avoid critical adverse events.
